# Do Progressive Intensities of Transcranial Direct Current Stimulation with and Without 40 Hz Binaural Beats Change Pre-Frontal Cortex Hemodynamics? A Randomized Controlled Trial

**DOI:** 10.3390/bs14111001

**Published:** 2024-10-27

**Authors:** Maria de Cassia Gomes Souza Macedo, Kariny Realino do Rosário Ferreira, Paula Almeida Meira, Arthur Ferreira Esquírio, Michelle Cristina Sales Almeida Barbosa, Gabriela Lopes Gama, Alexandre Wesley Carvalho Barbosa

**Affiliations:** Department of Physical Therapy, Laboratory of Non-Invasive Neuromodulation-LANN, Federal University of Juiz de Fora, Av. Moacir Paleta 1167, Governador Valadares 35010-180, MG, Brazil; mariacassia.macedo@estudante.ufjf.br (M.d.C.G.S.M.); kariny.realino@estudante.ufjf.br (K.R.d.R.F.); paula.almeida@estudante.ufjf.br (P.A.M.); arthurferreira.esquirio@estudante.ufjf.br (A.F.E.); michellecsalmeida@ufjf.br (M.C.S.A.B.); gabriela.gama@ufjf.br (G.L.G.)

**Keywords:** cognitive performance, binaural auditory beats, near-infrared spectroscopy, oxyhemoglobin, oxygen saturation, randomized controlled trial

## Abstract

Transcranial direct current stimulation (tDCS) is often reported to have positive effects on brain hemodynamics as well as cognitive performance. Binaural beats (BBs) have also shown the potential to improve cognitive performance. However, we could not find any studies assessing prefrontal hemodynamics using a combination of these techniques or assessing the effects on hemodynamic response at different intensity levels of tDCS (two and three mA). This study aimed to compare the immediate hemodynamic responses to tDCS at different intensities (two and three mA) with and without 40 Hz BBs. Sixty-eight healthy young individuals of both sexes were split into four groups: the tDCS 2 mA group; tDCS 3 mA group; tDCS 2 mA + BB group; and tDCS 3 mA + BB group. All groups received 20 min tDCS (F3-Fp2) alone or combined with BBs. The hemodynamic effect was assessed using a functional near-infrared intracranial spectroscope (fNIRS) positioned on the left supraorbital region (Fp1). The mean values of rates of oxygen saturation (SatO_2_) were recorded at baseline, during the intervention period, and post-stimulation. The oxygenated hemoglobin rates (HbO) were also extracted. No between-group differences were observed. The within-group analysis did not show significant differences in terms of the time×groups factor. However, the time factor showed significant within-group differences. No differences were found for the HbO rates. The present findings showed that two and three mA tDCS had effects on pre-frontal cortex SatO_2_; however, the use of additional BBs did not change the SatO_2_ levels compared to the use of tDCS alone.

## 1. Introduction

Transcranial direct current stimulation (tDCS) is a non-invasive neuromodulation technique that uses a low-intensity current (around one to two mA) to affect one’s excitability [1,2]. Studies have shown that tDCS can promote cognitive enhancement, such as increased working memory [3], inhibition (inhibitory control) [4], and cognitive flexibility [5]. While no serious adverse effects have been reported due to tDCS, mild headaches, skin redness, and itchiness of the skin under the electrodes are common complaints [6]. None of these symptoms are persistent though.

Two physiological mechanisms are responsible for the effects of tDCS: 1. shifts in the membrane resting potential threshold for depolarization or hyperpolarization according to anodic (positive) or cathodic (negative) montage [1,2,6]; and 2. increases in brain oxygenation and blood volume by stimulating specific glial cells (i.e., pericytes and astrocytes), due to the local increase in blood flow and vasodilation independent of neuronal metabolic activity [7,8].

Other studies show that deficits in executive functions are correlated with arterial brain oxygen desaturation, which impacts cognitive control, attention, and decision making [9,10]. Accordingly, the increases in intracranial oxygen saturation lead to neuromodulatory benefits on prefrontal executive functions [11]. The above-cited mechanisms generate a regional metabolic requirement that can be measured using functional near-infrared spectroscopy (fNIRS) [12,13].

A recent systematic review showed positive effects of tDCS on brain hemodynamics and cognitive performance [14]. The authors identified that these effects are most prominent in young adults. However, a moderate level of heterogeneity was determined for the application parameters, especially for tDCS intensity, which was limited to the usual intensities ranging from one to two mA [14].

Higher tDCS intensities have been more recently targeted for clinical purposes. A previous study proposed by Nitsche et al. (2017) assessed the safety and the tolerability of three and four mA tDCS. Interruption rules in case of severe adverse effects were set using tolerability questionnaires, skin temperature, and body resistance [15]. However, no major adverse events occurred, and the most frequent adverse effect was a transient skin redness without any skin damage in approximately 50% of the participants, suggesting the safety of intensities higher than two mA tDCS. A recent study showed that pre-frontal tDCS with an intensity of three mA had beneficial effects during emotional tasks [16]. Another study investigated the effect of different anodal tDCS intensities on motor sequence learning in older adults, including one, two, and three mA. The results suggested that the three mA intensity was more effective in certain motor tasks compared to lower intensities [17]. Despite these findings, evidence for intensities >two mA is still very limited.

There is also growing evidence that BBs can lead to a reduction in anxiety symptoms and improvements in cognitive performance [18]. The technique, first described by Heinrich Wilhelm Dove in 1839, consists of providing two distinct pure sinusoidal tones, one to each ear, with the same intensity and differences in their frequencies. The different frequencies lead to an illusory tone perception with a resultant equal to the average frequency of the two presented tones. Additionally, this illusory tone amplitude fluctuates at a frequency equal to the difference between the frequencies of the two tones [19]. For example, if frequencies of 400 Hz and 410 Hz are presented in the right and left ears, respectively, the perceived sound will have a frequency of 405 Hz which varies with an amplitude of 10 Hz. The process that leads to the integration of two tones with different frequencies to be perceived in a unified way is known as binaural integration [18,20]. A recent meta-analysis validated the applicability of BBs to increase cognitive performance [19], and frequently, beta and gamma frequencies have been associated with memory and attention, respectively. In addition, studies have found significant effects of using 40 Hz BBs to improve attention and memory [21,22]. Using the BB technique, another study identified the increased connectivity of neural networks on the prefrontal cortex using functional NIRS technology [23].

Despite the available evidence supporting the application of both techniques separately (tDCS and BBs), no study assessing prefrontal hemodynamics was found using the combination of these techniques. Also, no studies were found aiming to assess the effects of different levels of tDCS on hemodynamic response. Thus, the present study aimed to compare the immediate hemodynamic response to different intensities of tDCS (two and three mA) applied in isolation and combined with 40 Hz binaural beat (BBs).

## 2. Materials and Methods

### 2.1. Participants

**Figure 1:**
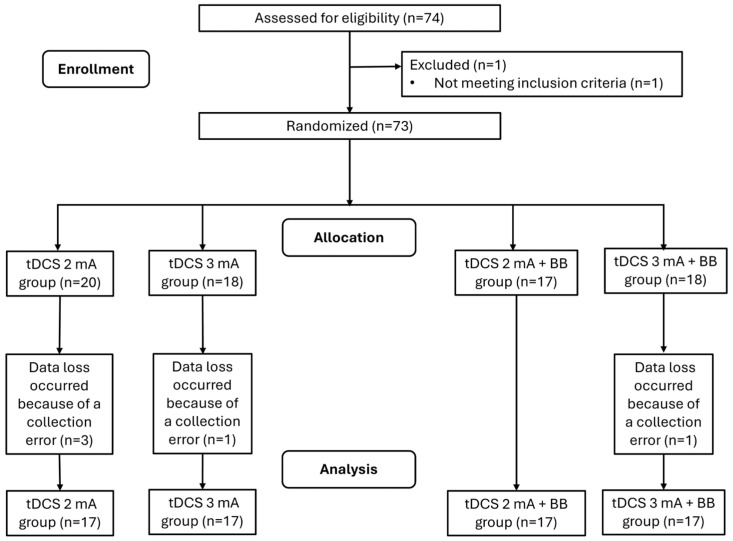
Flowchart of participant enrollment, allocation, and analysis in accordance with CONSORT guidelines.

Sixty-eight healthy young participants of both sexes (18–35 years) were selected to join the present study. An a priori one-tailed sample size calculation was performed using the G-Power software (version 3.1.5, Franz Faul, Universität Kiel, Germany) [24,25], with an α level of 0.05, an expected power (1 − β) of 0.80, and an effect size of 0.63, which was obtained from a previous study [14]. The returned sample size was composed of participants. However, considering a 30% sample loss, the final sample consisted of 68 participants. The inclusion criteria were as follows: participants who were between 18 and 30 years old, right-handed, who did not have impairments in auditory acuity, who had no history of neuropsychiatric disorders, and who did not self-report the use of any psychoactive substances. The exclusion criteria were the self-reported absence of the following conditions: cardiac pacemakers, pregnancy, scalp lesions, and metals in or near the head (e.g., cochlear implants, aneurysm clips or coils, firearm projectile fragments, jewelry, and hair clips). After the screening, the participants were randomly assigned to 1 of 4 groups: tDCS 2 mA group, receiving tDCS at 2 mA; tDCS 3 mA group, receiving tDCS at 3 mA; tDCS 2 mA + BB group, receiving tDCS at 2 mA plus BB; and tDCS 3 mA + BB group, receiving tDCS at 3 mA plus BB, as shown in Figure 1. There was no sham group, as each technique’s efficacy had already been proven [14,18]. The randomization was carried out by an independent rater considering an allocation ratio of 1:1:1:1. Before the study began, a random allocation sequence was automatically generated using the Research Randomizer website (www.randomizer.org, accessed on 7 September 2023), by using 1 set of numbers, with a total of 68 numbers per set, and the established number range was 1–4, representing the group. The random sequence was generated by the Research Randomizer, and the independent rater followed the sequence. The sequence order was continuously given to the examiner who performed the assessments when a new participant was allocated for treatment. The rater who performed the randomization was blinded to the statistical analysis. The allocation concealment was preserved by informing the therapist of the participant’s group assignment only after their enrollment in the research.

This randomized clinical trial was conducted according to the Declaration of Helsinki. The Ethics Committee of the Federal University of Juiz de Fora (number 69344723.3.0000.5147) approved all the procedures employed in the present study. The trial was registered in the Brazilian clinical trials registry (number RBR-2kg65g7). Participants were informed about the assessments, interventions, risks, and benefits of the present study. All participants signed the informed consent form.

### 2.2. Transcranial Direct Current Stimulation (tDCS)

Electrical stimulation was applied using an analog device (Neuroeletrical, São Paulo, Brasil) with a maximum current of 4 mA. The applied current intensity was 2 mA or 3 mA, for 20 min. Two 35 cm^2^ (5 × 7 cm) rubber electrodes wrapped in a saline solution-soaked sponge were utilized to apply the tDCS. A distance of at least 7 cm was maintained between electrodes to avoid the shunting effect [1]. The electrode placement was according to the international 10–20 system. The anode was positioned on F3 and the cathode on Fp2 for the left dorsolateral prefrontal cortex (DLPFC) and right supraorbital region, respectively. A 30 s ramp-up at the beginning and a 30 s ramp-down at the end of the stimulation period were used.

### 2.3. Binaural Beat Stimuli

The stimuli consisted of 300 Hz and 340 Hz waves on the left and right ear, respectively, resulting in a 40 Hz BB (gamma frequency). The BB was embedded in white noise and controlled by the Brain Waves App (version 8.1.0). The stimuli were applied using on-ear headphones for 20 min, simultaneously with tDCS.

### 2.4. fNIRS Recording

To assess the effects of tDCS and BBs upon brain cortical activity, a functional near-infrared intracranial spectroscopy (fNIRS) system (Humon Hex, Dynometrics Inc., Boston, MA, EUA) was used. The system utilized near-infrared light at two specific wavelengths (660 nm and 850 nm) to collect raw fNIRS data. The brain signals were then transmitted wirelessly to a laptop, which was used to process the optical intensity measurements and convert them into changes in concentrations of oxygenated and deoxygenated hemoglobin (ΔHbO/ΔHbR) using the modified Beer–Lambert law. The device was connected through Bluetooth to a smartphone (MoxZones, Dynometrics Inc., Boston, MA, EUA, retrieved from https://moxzones.com/, accessed on 11 September 2023), showing the real-time O_2_ saturation level as a percentage (SatO_2_). The fNIRS infrared light emitters and sensors measure the differential absorption of near-infrared light between oxyhemoglobin (HbO) and deoxyhemoglobin (HbR). Studies have shown that oxygenated hemoglobin (HbO) has a better signal-to-noise ratio and greater sensitivity to changes in blood flow compared to deoxyhemoglobin (HbR) [26]. For this reason, the focus of this study was on HbO concentration.

### 2.5. Experimental Protocol

The fNIRS sensor was positioned on the Fp1 region, and then electrodes for tDCS application were positioned and fixed with a headband. To avoid between-subject variability and to provide reliable positioning of the fNIRS device, all head measures were according to the 10–20 international system [27]. We followed the next steps, which are detailed as follows. Step 1: Identifying the reference points—locate the nasion, which is the indentation between the forehead and the nose; locate the inion, the bony prominence at the lower rear part of the skull; and identify the preauricular points, which are the indentations just in front of the ears on both sides. Step 2: Measuring the head circumference—using a measuring tape, measure the circumference of the head from the nasion to the inion, passing just above the ears. Step 3: Determining the midline points—measure the distance from the nasion to the inion and mark the midpoint along this line with a marker (this is the Cz position). Step 4: Marking the Fp1 position—measure 10% of the nasion–inion distance up from the nasion along the midline to locate the Fpz position (frontal pole midline). From Fpz, measure 10% of the total head circumference laterally towards the left side of the head to locate Fp1. Step 5: Marking the Fp2 position—measure 10% of the nasion–inion distance up from the nasion along the midline to locate the Fpz position (frontal pole midline). From Fpz, measure 10% of the total head circumference laterally towards the right side of the head to locate Fp2. Step 6: Marking the F3 position—from Fpz, measure 20% of the total head circumference laterally towards the left side of the head to locate F7. From F7, measure 25% of the total nasion–inion distance along the line connecting F7 to Cz (central point on the midline of the scalp) to locate F3. The tDCS 2 mA + BB and tDCS 3 mA + BB groups had in-ear headsets, which were also positioned. After positioning, the fNIRS sensor recorded the baseline SatO_2_. After 2 min (baseline), the intervention was applied for each group as previously described. After the 20 min intervention, the fNIRS continued collecting data for another 2 min (post-stimulation period). The assessment and the intervention protocols were carried out in a private room.

### 2.6. Data Extraction

**Figure 2:**
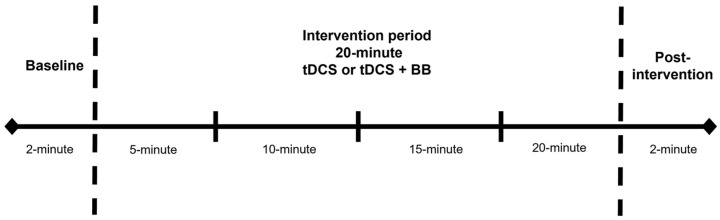
The experimental timeline showing six intervals: baseline (2 min); intervention period (20 min, 4 × 5 min epochs); post-intervention (2 min).

The mean levels of SatO_2_ and HbO obtained during the experimental protocol were recorded for each participant. The overall experiment was split into 6 intervals: baseline (2 min); 20 min intervention period subdivided into 4 × 5 min epochs at 5 min, 10 min, 15 min, and 20 min; and post-intervention (2 min), as shown in Figure 2.

### 2.7. Statistical Analysis

The mean SatO_2_ and HbO levels were analyzed for each interval. The Shapiro–Wilk test and Levene’s test were used to assess data normality and homogeneity, respectively. A factorial analysis of variance with repeated measures was used to identify between- and within-group differences. When necessary, Bonferroni’s post hoc correction test was used for pairwise comparisons, avoiding multiple comparisons. The magnitude of the η_2p_ was qualitatively interpreted using the following thresholds: ~0.01 (small), ~0.06 (medium), and ~0.14 (large) [28]. The significance was set at *p* < 0.05. The Jamovi software (the Jamovi project (2022), v. 2.3, retrieved from: https://www.jamovi.org, accessed on 1 July 2022) was used for all statistical analyses, with a significance level of *p* < 0.05.

## 3. Results

According to the sample size calculation, 17 participants were allocated to each group (n = 68 participants). A total of 74 participants were recruited, but one did not meet the inclusion criteria. Three participants were excluded from the tDCS 2 mA group, one participant was excluded from the tDCS 3 mA group, and one participant was excluded from the tDCS 3 mA + BB group, as shown in Figure 1. Another five participants were then allocated to reach the minimal sample size. Participants were recruited from September to December 2023. The participants’ characteristics are shown in Table 1. The mean and standard deviation values of SatO_2_ are shown in Table 2. No significant between-group differences were found (F = 0.123; *p* = 0.946; η_2p_= 0.006 (small)). The within-group analysis did not show any significant differences in terms of the time×group factor (F = 0.837; *p* = 0.636; η_2p_ = 0.038 (small)). However, significant differences were noted in the within-group analysis in terms of the time factor (F = 7.058; *p* = 0.001; η_2p_ = 0.101 (medium)). Significant differences were found between the baseline results and the results at all of the other moments (baseline vs. 5 min: *p* = 0.02; baseline vs. 10 min: *p* = 0.04; baseline vs. 15 min: *p* = 0.03; baseline vs. 20 min: *p* = 0.01; baseline vs. post-stimulation: *p* = 0.008). The HbO analysis did not show any significant differences for within-group factors (time [F = 0.934; *p* = 0.459; η_2p_ = 0.014 (small)]; time×group [F = 1.284; *p* = 0.210; η_2p_ = 0.057 (small)]) or between-group factors (F = 0.340; *p* = 0.797; η_2p_ = 0.016 (small)), as shown in Table 3.

## 4. Discussion

The present study tested the hypothesis that tDCS stimulation with BBs would provoke left DLPFC activation and consequently increase SatO_2_ and HbO levels. The SatO_2_ values showed no significant between-group differences. Also, there were no within-group differences considering the time×group factor. However, within-group time factor differences were observed between the baseline results and the results for all other intervention moments and the post-intervention period. The HbO values showed no within- or between-group differences. 

Preclinical studies have identified that tDCS can lead to vasodilation and increased cerebral blood flow [29,30]. tDCS can increase neuronal membrane potential at rest, leading to an increase in neuronal activity, and requiring brain perfusion-mediated neurovascular coupling adaptations [14]. A study using functional magnetic resonance imaging analyzed the effects of 2 mA tDCS stimulation applied to the primary motor cortex on cerebral blood flow [31]. The above-mentioned study showed increased blood flow compared to that of the sham stimulation group. These findings partially corroborate the results of the present study, which showed an increase in the SatO_2_ levels in the within-group comparison for all of the intervention groups. However, no between-group differences were noted.

The findings of other studies differ from these findings [32,33]. A study assessed the effects of anodal tDCS in patients with mild traumatic brain injury [32]. The authors identified a decrease in cerebral oxygenation values. Another study applied anodal tDCS at different intensity ranges (0.5, 1.0, 1.5, and 2.0 mA) over the primary motor cortex, but the results showed inconsistent increases in cerebral blood flow [33].

HbO levels were not significant at all. Similar findings were recorded in a study with 61 healthy young adults randomly assigned to one of the three groups: the sham treatment, left anodal/right cathodal tDCS, and left cathodal/ right anodal tDCS [34,35]. The electrodes were placed on F3 and F4 with 1.0 mA tDCS for 25 min. No significant differences were found for HbO values measured on the frontotemporal and dorsolateral prefrontal cortex [34]. Another study found an HbO decrease when stimulating the prefrontal cortices of young people bilaterally for 15 min using one mA [35]. On the other hand, a study identified increased HbO levels using anodal 1.5 mA tDCS for 10 min on the left prefrontal cortex of 24 young adults [36]. Another study with 21 older adults showed an increase in the HbO levels on the left frontal area using anodic 1.5 mA tDCS for 26 min [12]. The overall HbO results’ heterogeneity may be explained by the employment of distinct cognitive tasks in combination with tDCS. The current study avoided any combined tasks along with the tDCS, so the singularity of tDCS stimulation would be preserved, and the cognitive arousal would not affect the hemodynamic outcome [7,8]. 

The adjustment of tDCS parameters seems to change the physiological and psychological outcomes [15]. A key parameter is the current’s intensity, as higher intensities seem to be more effective [37]. The present study assessed the effects of two and three mA tDCS on brain hemodynamic responses. However, no significant differences were noted in the between-group analysis. The results showed that using an intensity of three mA did not provide additional benefits in terms of the cerebral oxygenation variables. These findings can be explained by a “ceiling” effect in healthy people. As they already had an adequate basal oxygenation level, little room was left for improvements in cerebral oxygenation. Therefore, higher intensities may not be an effective parameter for altering brain hemodynamics.. In addition, considering that using intensities greater than two mA may not bring greater benefits and that most available tDCS devices only provide currents up to two mA [38], the need for devices with higher intensities is arguable.

Previous studies have shown that BBs can change mental states [16,17]. BBs are based on the brainwave entrainment hypothesis, which suggests that auditory stimulation at a specific frequency leads to the electrocortical activity of the brain oscillating at the same frequency as the external signal [39]. According to this hypothesis, brain stimulation using a 40 Hz BB could provoke neurophysiological effects, increasing the activation of the prefrontal cortex and consequently improving cognitive performance [18,19]. In a previous study, 36 young adults were randomly divided into two groups: 18 participants listened to 40 Hz BBs and 18 participants listened to a constant 340 Hz tone (the control condition) for 3 min before and during a cognitive task [40]. The results of the study showed that the BB group had better focused visual attention than those in the control group [40]. A systematic review showed that tDCS can also modulate cortical activity, which results in improved cognitive performance and increased cerebral oxygen hemodynamics [14]. The current study hypothesized that the combination of BB with tDCS of two or three mA could result in a synergistic effect, which would lead to increased cerebral blood flow, assessed through changes in the SatO_2_ in DLPFC. Specifically, it was expected that the techniques’ combination would significantly increase cerebral oxygenation compared to tDCS alone. In addition, greater effects were expected for the 3 mA tDCS group plus BB. However, this hypothesis was not confirmed, as the BBs did not change the cerebral oxygenation levels in any group. Although both techniques may individually lead to increased cortical activity, the present findings suggest that their combination may not have been enough to provoke a significant increase in cerebral oxygenation during the tested conditions.

The current study highlights the physiological impact of tDC and BBs on cerebral hemodynamics, but no significant between-group or within-group differences were found in the core metrics of oxygenated hemoglobin (HbO) and oxygen saturation (SatO_2_). This outcome suggests several key points for future research and clinical applications. While other studies have suggested that increasing the tDCS intensity above two mA might improve cognitive performance and blood flow, this study found no significant hemodynamic advantages at three mA in healthy participants. This finding points to a “ceiling effect”, in which individuals with normal baseline oxygenation may not experience further benefits from higher stimulation intensities. Future research should examine whether this ceiling applies to other populations, such as individuals with impaired oxygenation or neurological disorders, and whether long-term tDCS application produces different results. Additionally, despite prior evidence suggesting that both tDCS and BBs individually improve cognitive functions and cortical activation, their combination did not show a synergistic effect in this study. Future investigations should explore different combinations of these techniques, possibly varying parameters such as stimulation duration or performing cognitive tasks during stimulation. Additionally, populations with pre-existing cognitive impairments or hypoxic conditions might respond differently to the combined intervention. Finally, a limitation identified in this study was the short post-intervention measurement period (2 min), which may not have been sufficient to capture delayed hemodynamic effects. Extending this post-intervention period could reveal more substantial changes in oxygen saturation and HbO levels. Moreover, including other populations (e.g., older adults or clinical groups) may yield different insights, particularly in cases in which their baseline hemodynamic levels are lower, leaving more room for improvements through intervention. 

The results of this study hold important clinical implications, particularly in neuromodulation therapy using tDCS for improving cerebral oxygenation. The study confirmed that both two mA and three mA tDCS intensities can increase oxygen saturation (SatO_2_) in the prefrontal cortex, affirming the potential of tDCS as a non-invasive tool to influence brain hemodynamics. This has practical relevance for clinical settings in which enhancing cerebral blood flow and oxygenation could benefit patients with conditions affecting their cognitive functions, such as those with mild cognitive impairments or those undergoing post-stroke rehabilitation. While the combination of tDCS with 40 Hz binaural beats (BBs) did not significantly enhance the effects compared to tDCS alone, this finding is clinically useful. It suggests that tDCS alone, even at the standard two mA intensity, may be sufficient for interventions targeting brain oxygenation in healthy individuals. This insight is valuable for practitioners aiming to optimize treatment protocols, reducing the need for additional interventions like BBs, which may simplify the treatment approach in clinical settings. Moreover, this study reinforces the safety of applying tDCS at intensities up to three mA, an important consideration for clinicians seeking to use higher intensities without adverse effects. The absence of significant hemodynamic differences between two mA and three mA suggests that higher intensities may not be necessary in healthy populations, though this could vary in clinical populations with impaired cerebral hemodynamics. As such, future studies might focus on patients with neurovascular conditions, for whom higher tDCS intensities could prove more beneficial.

Some limitations must be addressed. The present study assessed the immediate effects of tDCS in combination with BBs. However, more sessions might be necessary to find consistent results. However, other studies assessing the immediate effects are needed to primarily test new protocols and support further prospective investigations. Another limitation was the SatO_2_ assessment time (~2 min) post-stimulation. A longer assessment time would show significant between-group changes in SatO_2_, allowing for a more comprehensive analysis. Further studies might consider longer post-intervention assessments. The present study sample was composed of healthy young people. Further studies should test the same protocol in other populations. Those with neurological and psychiatric disorders may show distinct outcomes in different clinical contexts.

## 5. Conclusions

The present study showed that the combination of BBs and tDCS did not significantly influence SatO_2_ levels compared to tDCS alone. However, tDCS at an intensity of two mA and three mA seems to indistinctly influence brain oxygenation. Further research is needed to explore the long-term effects of combining both techniques, especially in people with neurological or psychiatric disorders, as they are often affected by their brain hemodynamics.

## Figures and Tables

**Table 1:** Participants’ characteristics.

Characteristic	tDCS 2 mA		tDCS 3 mA		tDCS 2 mA + BB		tDCS 3 mA + BB		*p* Value
	Mean ± SD	n (%)	Mean ± SD	n (%)	Mean ± SD	n (%)	Mean ± SD	n (%)	
Sex									0.49 *
Male		6 (35.30)		3 (17.65)		5 (29.41)		7 (41.18)	
Female		11 (64.70)		14 (82.35)		12 (70.59)		10 (58.82)	
Age (years)	23.5 ± 2.65		25.6 ± 7.56		23.01 ± 3.05		22.9 ± 2.88		0.44
Weight (kg)	68.0 ± 15.8		66.0 ± 9.97		70.3 ± 13.1		70.7 ± 19.2		0.26
Height (m)	1.66 ± 0.06		1.66 ± 0.10		1.69 ± 0.08		1.70 ± 0.06		0.29
(BMI)	24.7 ± 4.59		24.1 ± 3.40		24.9 ± 5.12		24.4 ± 6.14		0.53

Legend: tDCS = Transcranial direct current stimulation; BB = binaural beat; BMI = body mass index. * Chi-square test.

**Table 2:** Oxygen saturation (SatO_2_) level results during interventions as shown as %.

Group	Baseline	5 min	10 min	15 min	20 min	Post-Stimulation
tDCS 2 mA	54.3 ± 6.77	54.4 ± 6.43	55 ± 6.67	54.7 ± 6.66	54.3 ± 6.94	55 ± 7.30
tDCS 3 mA	55 ± 6.83	56.3 ± 7.12	56.1 ± 7.12	56.3 ± 7.24	56.2 ± 7.52	56.5 ± 7.65
tDCS 2 mA + BB	53.6 ± 8.23	54.3 ± 8.95	54.4 ± 8.91	54.7 ± 8.69	55 ± 8.59	54.9 ± 8.34
tDCS 3 mA + BB	54 ± 7.24	54.6 ± 7.12	54.6 ± 6.95	54.9 ± 6.96	55.2 ± 6.99	54.8 ± 6.67

Legend: tDCS = Transcranial direct current stimulation; BB = binaural beat.

**Table 3:** HbO level results during interventions in g/dL.

Group	Baseline	5 min	10 min	15 min	20 min	Post-Stimulation
tDCS 2 mA	6.1 ± 1.03	6.11 ± 0.98	6.18 ± 1	6.13 ± 0.985	6.07 ± 1.02	6.17 ± 1.08
tDCS 3 mA	6.32 ± 1.08	6.56 ± 1.11	6.53 ± 1.1	6.53 ± 1.1	6.06 ± 1.93	6.1 ± 1.95
tDCS 2 mA + BB	5.83 ± 1.17	5.93 ± 1.33	5.92 ± 1.31	5.98 ± 1.29	6.06 ± 1.29	6.06 ± 1.24
tDCS 3 mA + BB	6.04 ± 1.13	6.15 ± 1.11	6.19 ± 1.08	6.23 ± 1.08	6.26 ± 1.10	6.18 ± 1.05

Legend: tDCS = Transcranial direct current stimulation; BB = binaural beats.

## Data Availability

The raw data are fully available online [41].
